# Robot-assisted radical prostatectomy in a patient with prostate cancer complicated by benign prostate hypertrophy with middle lobe hypertrophy

**DOI:** 10.1093/jscr/rjae077

**Published:** 2024-02-21

**Authors:** Yoshiaki Kawamura, Takato Uchida, Tatsuya Umemoto, Nobuyuki Nakajima, Masahiro Nitta, Masanori Hasegawa, Sunao Shoji, Akira Miyajima

**Affiliations:** Department of Urology, Tokai University School of Medicine, 143 Shimokasuya, Isehara city, Kanagawa 259-1193, Japan; Department of Urology, Tokai University School of Medicine, 143 Shimokasuya, Isehara city, Kanagawa 259-1193, Japan; Department of Urology, Tokai University School of Medicine, 143 Shimokasuya, Isehara city, Kanagawa 259-1193, Japan; Department of Urology, Tokai University School of Medicine, 143 Shimokasuya, Isehara city, Kanagawa 259-1193, Japan; Department of Urology, Tokai University School of Medicine, 143 Shimokasuya, Isehara city, Kanagawa 259-1193, Japan; Department of Urology, Tokai University School of Medicine, 143 Shimokasuya, Isehara city, Kanagawa 259-1193, Japan; Department of Urology, Tokai University School of Medicine, 143 Shimokasuya, Isehara city, Kanagawa 259-1193, Japan; Department of Urology, Tokai University School of Medicine, 143 Shimokasuya, Isehara city, Kanagawa 259-1193, Japan

**Keywords:** robot-assisted radical prostatectomy, benign prostatic hyperplasia, prostate cancer, middle-lobe hypertrophy

## Abstract

Robot-assisted radical prostatectomy (RARP) is difficult in patients with benign prostatic hyperplasia (BPH), a condition causing frequent urination, because of the large prostate volume and particularly true when BPH is accompanied by an enlarged middle lobe. To overcome this difficulty, some surgeons elevate the middle lobe with a third arm or tow the urethral catheter to the edge to identify the resection line. Herein, we describe a method for lifting a prostate with an enlarged middle lobe, which was successfully applied in a patient with prostate cancer and BPH. This technique can help identify the resection line between the bladder and prostate, reducing surgical difficulty and the number of unnecessary sutures.

## Introduction

Robot-assisted radical prostatectomy (RARP) is a widely accepted standard treatment for localized prostate cancer. RARP was first approved for insurance coverage in Japan in April 2012 and has since been widely used for localized prostate cancer [1]. This widespread use of RARP implies that there are more opportunities for surgery and a greater risk of complications. Various factors have been reported to increase surgical difficulty, including body mass index, working space, and prostate volume [2, 3]. Benign prostatic hyperplasia (BPH) is a common urological disorder that induces dysuria [4]. However, prostate carcinoma is often associated with BPH, which can increase the difficulty of surgery. This is particularly true in cases of middle-lobe hypertrophy. In this report, we present a possible approach for addressing such cases.

## Case report

**Figure 1:**
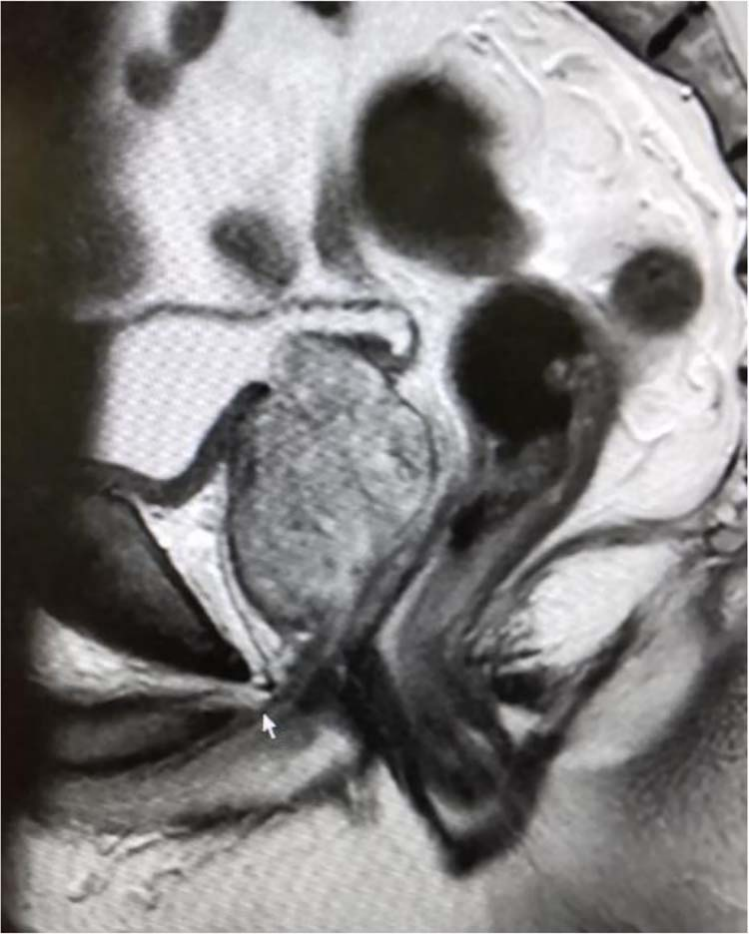
Magnetic resonance imaging before surgery showing BPH with middle lobe hypertrophy.

The patient was a 72-year-old man with BPH, a prostate volume of 50.0 ml, and high middle-lobe hypertrophy (Fig. 1). His initial prostate-specific antigen level was 14.94 ng/ml, and preoperative magnetic resonance imaging revealed PI-RADS III in the left peripheral zone. Prostate biopsy was 1/10 positive and revealed adenocarcinoma with a Gleason score of 4 + 4 = 8 (cancer core length 5/15 mm) in the left peripheral zone. Following the biopsy, the patient had a urethral catheter inserted as he could not urinate due to the BPH. The patient had a history of hypertension, true polycythemia vera, and cerebral infarction; further, he had an Eastern Cooperative Oncology Group performance status of 0 and was taken off antiplatelet medications before surgery.

**Figure 2:**
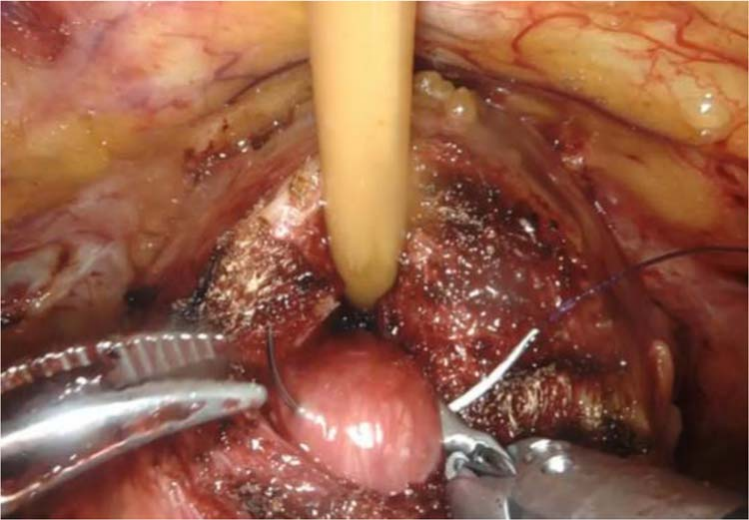
Puncture of the middle-lobe using a 25-cm, 26-mm, 3–0 monofilament needle.

**Figure 3:**
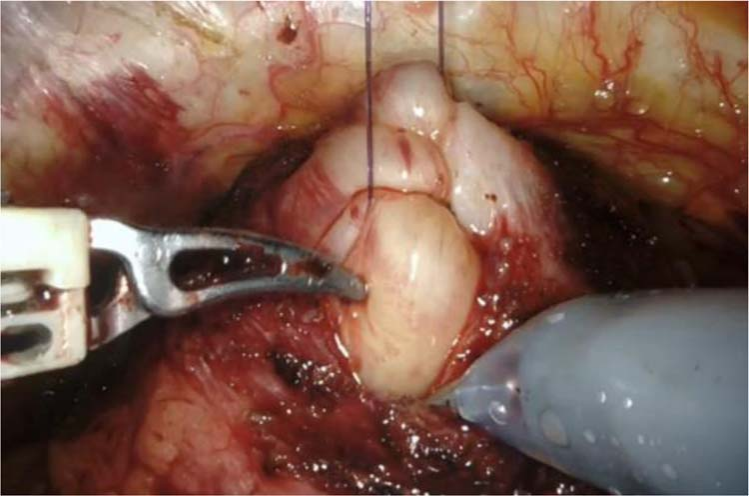
Grasping of the thread with the fourth arm followed by lifting.

RARP was performed without preoperative hormone therapy. All steps of the RARP procedure were based on a previously established method described by Patel *et al.* [5], in which all procedures were performed via the transperitoneal approach with a six-port technique. In our patient, we performed conventional RARP. In cases with middle-lobe hypertrophy, prostate dissection around the bladder neck is an important feature. In this procedure, the border between the bladder neck and prostate is dissected using electrocoagulation, and when the middle lobe of the prostate is exposed, it is punctured using a 25-cm, 26-mm, 3–0 monofilament needle and lifted using the fourth arm several times (Fig. 2). The manual procedure, involving pulling in various directions and re-puncturing when the middle lobe is exposed, is repeated until the middle lobe is completely exposed. This reveals the bladder trigone and ureteral orifice and allows identification of the appropriate resection line. At this point of the dissection, the prostate is dissected, the dorsal seminal vesicle is penetrated, and finally, the Denonvilliers fascia is dissected (Fig. 3). Surgery was completed without additional suturing in the bladder neck side. The console time was 2 h 9 min, and the anesthesia time was 3 h 40 min. Bleeding volume was 28.5 ml, including urine volume. The weight of the removed prostate was 70 g. The pathological features were as follows: tumor size 10 × 5 mm (largest tumor layer); Gleason score 4 + 3 = 7; and adenocarcinoma pT2c, ly0, v0, pn0, sv0, EPE0, and RM0. Postoperatively, there were no anastomotic leaks, and no serious complications, categorized as grade 2 based on the Clavien–Dindo classification v2.0, developed.

## Discussion

Surgical treatment of patients with concomitant BPH and prostate cancer is often challenging because of increased surgical difficulty. It is common for patients to request surgery after treatment with dutasteride and endocrine therapy to lower the surgical impact; however, there are times when surgery must proceed based on the patient’s wishes and other factors. In some cases, when an incision is made in the prostatic area, the bladder neck may be opened too wide, and a large middle lobe may hang down and obstruct the view when the seminal vesicle and ductus deferens are excised, or the Denonvilliers fascia is removed, thereby increasing the risk of rectal injury. Our technique of lifting the prostate using sutures may reduce the extent of suturing and the risk of rectal injury and contribute to the observed decrease in postoperative complications due to BPH [6]. We hope that these measures will help ensure a safe and reliable surgery when RARP becomes more widespread.

In conclusion, suspension of the enlarged middle lobe can help identify the incision plane for prostate dissection around the bladder neck, allowing safe performance of RARP in patients with prostate cancer and BPH.
